# In vivo validation of osteoinductivity and biocompatibility of BMP-2 enriched calcium phosphate cement alongside retrospective description of its clinical adverse events

**DOI:** 10.1186/s40729-024-00567-6

**Published:** 2024-10-30

**Authors:** Mingjie Wang, Chunfeng Xu, Yuanna Zheng, Herman Pieterse, Zhe Sun, Yuelian Liu

**Affiliations:** 1 Department of Oral Cell Biology, Academic Centre for Dentistry Amsterdam (ACTA), Vrije Universiteit Amsterdam and University of Amsterdam, Amsterdam, The Netherlands; 2 Department of Second Dental Center, Shanghai Jiao Tong University School of Medicine, College of Stomatology, Shanghai Jiao Tong University, National Centre for Stomatology, National Clinical Research Center for Oral Diseases, Shanghai Key Laboratory of Stomatology, Shanghai Research Institute of Stomatology, Shanghai, China; 3 Ningbo Dental Hospital, Ningbo Oral Health Research Institute, Ningbo, Zhejiang China; 4 School/Hospital of Stomatology, Zhejiang Chinese Medical University, Hangzhou, Zhejiang China; 5 Heymans Institute of Pharmacology at Ghent University, Ghent, Belgium; 6 Profess Medical Consultancy B.V., Heerhugowaard, The Netherlands

**Keywords:** BMP-2, Calcium phosphate cement, Maxillofacial surgery, Adverse events

## Abstract

**Purpose:**

Although bone morphogenetic protein-2 (BMP-2) possesses potent osteoinductivity, there have been some concerns on the safety of BMP-2 and BMP-2-incorporated bone substitutes used for bone formation. On the other hand, BMP-2-loaded calcium phosphate cement (BMP-2@CPC) has been developed and used for bone regeneration in oral implantology. Therefore, this study aims to investigate this product's biocompatibility and clinical safety after being used in maxillofacial surgery.

**Materials and methods:**

A rat model was employed to assess the osteoinduction and biocompatibility of BMP-2@CPC. Further, a retrospective investigation was carried out: 110 patients who received BMP-2@CPC treatment after their maxillofacial surgery were recruited to describe relative adverse events.

**Results:**

In vivo, BMP-2@CPC showed a significantly higher mean bone volume density and osteoblasts volume density (15 ± 2% and 3 ± 1%)than those of the CPC group (*p* < 0.05) after being implanted in the dorsal area of rats. Regarding biocompatibility, the mean fibrous tissue volume density was significantly lower in the BMP-2@CPC group (20 ± 5% compared to 31 ± 6%, *p* = 0.026). The retrospective clinical study showed that only five mild/moderate adverse events were identified in four patients based on the medical records of 110 patients, including swelling, bony mass, and wound dehiscence. This adverse event occurrence was not affected by gender, age, the dose of filled materials, and operations in the study (*p* > 0.05).

**Conclusions:**

BMP-2-loaded CPC has osteoinductivity and more promising biocompatibility than pure CPC. However, its degradation is slower than CPC. The safety of BMP-2-loaded CPC with 0.5 or 1 mg BMP-2 is promising in oral maxillofacial surgery.

**Clinical implications:**

This study confirmed the promising safety of this BMP-2 incorporated CPC used in dental clinical practice, which can promote its reassuring application for dental implant placement in bone insufficient areas.

**Graphical Abstract:**

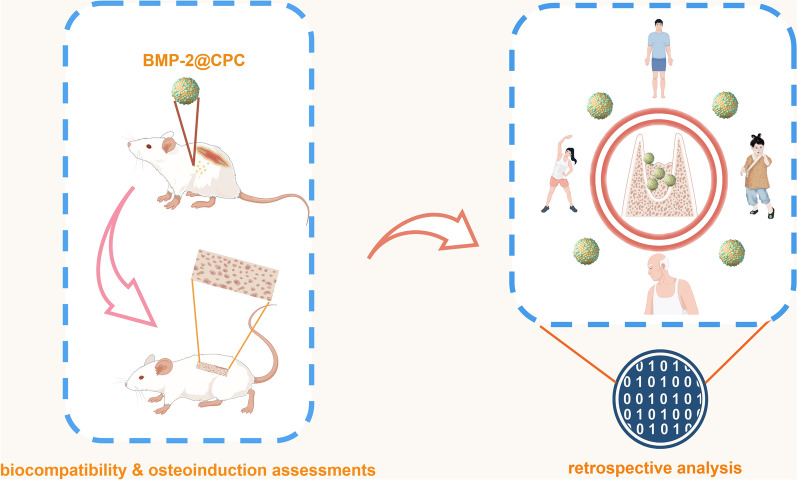

**Supplementary Information:**

The online version contains supplementary material available at 10.1186/s40729-024-00567-6.

## Introduction

Bone defects in the jaw caused by infection, trauma, or tumor resection are constant challenges for dental implant placement [1]. Autologous bone is widely used to repair these bone defects and is considered the “gold standard” due to its excellent osteoinductivity and osteoconductivity [2]. Nevertheless, its application is limited by some drawbacks, including extra surgery for bone collection, morbidities of donor sites, limited bone volume, and fast resorption [3].

To eschew these inherent disadvantages of autografts, calcium phosphate (CaP) biomaterials, for instance, hydroxyapatite, beta-tricalcium phosphate, and calcium phosphate cement (CPC), have been synthesized and used as alternatives to autografts as they have extensive source, reliable quality, and potential of combining with therapeutic molecules for tissue engineering [4]. CPC, a mixture of tetracalcium phosphate and dicalcium phosphate anhydrous, has been used in long bone and spine fracture repair in patients [5, 6] and received growing attention due to the easily-shaped capability, ideal biocompatibility [7], and biomechanically stability [8]. At the cellular level, it has been testified that CPC is beneficial for cell attachment and osteoblast phenotype expression [9]. However, as with other CaP bone substitutes, CPC cannot induce the osteogenic differentiation of stem cells, i.e. lacking osteoinductivity, weakening its therapeutic efficacy in bone regeneration.

Growth factors that can stimulate osteogenic differentiation of stem cells can be used to refine CPC, thereby improving its treatment efficiency. Bone morphogenetic protein-2 (BMP-2), a bone growth factor, is one of the TGF-beta family members. It has been well-known for its potent osteoinductivity [10] and has been introduced into clinical applications. This growth factor was incorporated into various bone substitutes in clinical settings as a potential alternative to autologous bone. In 2007, a BMP-2-loaded collagen sponge was approved by the FDA for orthopedic and dental applications [11–13], and achieved promising clinical success.

Similarly, loading BMP-2 into CPC (BMP-2@CPC) has been reported for osteoinductivity, thereby enhancing bone defect repair [9]. However, concerns on the side effects of BMP-2, including infection, ectopic bone formation, soft tissue swelling, and even tumorigenesis, have been raised, and most of these BMP-2-associated side effects are linked to its supraphysiological concentration as a result of its rapid release from the delivery system [14, 15]. Unfortunately, some side effects after BMP-2 application in maxillofacial surgery, such as edema, erythema [16], severe soft tissue swelling, wound dehiscence [17], and potential tumorigenicity [18], have also been reported. Therefore, the concerns on the safety of BMP-2@CPC in dental implantology have increased.

In the present study, an in vivo rat study was conducted to evaluate the osteoinductivity and biocompatibility of BMP-2@CPC. Afterward, we evaluated BMP-2@CPC's performance in the maxillofacial application using a retrospective investigation, in which data from recruited patients were analyzed two years after receiving treatment. We trust that we can create a safety profile for the BMP-2@CPC application in maxillofacial surgery. Results will be used as the starting point to build an adverse events report system to fulfil the mandatory post-market surveillance of the EU MDR 2017/745 and US FDA requirements.

## Material and methods

### Preparation of CPC granules

The CPC granules used in this study are Chitai^®^ (Shanghai Rebone Biomaterial, Shanghai, China). Briefly, they were synthesized by combining dicalcium phosphate anhydrous (DCPA) with equimolar tetra calcium phosphate (TECP). To obtain the high-purity TECP to achieve a solid-to-solid reaction between calcium phosphate and calcium carbonate, the temperature was set at 1500 degrees Celsius for 8 h. Ammonium hydrogen phosphate [(NH_4_)_2_HPO_4_] and calcium nitrate [Ca (NO_3_)_2_] were set at an acidic pH to prepare Dicalcium phosphate dihydrate (DCPD, CaH- PO_4_^.^2H_2_O). DCPA was obtained by evaporating the crystallization water in DCPD at 120 degrees Celsius for 5 h. The scaffolds were prepared at a specific ratio of TECP: DCPA (73.21: 26.79 in weight, aiming to have a 1.67 Calcium/ Phosphor molar ratio to achieve the same ratio of bone tissue), in which NaCl granules ~ 500 μm in size were incorporated and combined with 100 μL saturated NaCl solution in water to form the cement mire. The mire was then cast into a mold under the pressure of 2 MPa for one minute, to avoid H_2_O loss at the high pressure or granule deformation at low pressure. Then the granules were prepared into 0.25 to 1 mm CPC-granule samples weighing 0.25 g each, which will be implanted in the dorsal sites of the rats.

### Preparation of BMP-2@CPC granules

The abovementioned CPC granules were sterilized by ethylene oxide vapor. Recombinant human BMP-2(rhBMP-2, Shanghai Rebone Biomaterial, Shanghai, China) in acetic acid solution was doped onto CPC granules and stayed 4 h until fully absorbed. Afterward, the granules were lyophilized and stored at − 20 degrees Celsius for later use. The final amount of rhBMP-2 loaded in CPC is about 1 mg of rhBMP-2 in 1 g CPC.

### Topography characterization of CPC and BMP-2@CPC

The BMP-2@CPC granules were sputter-coated with gold particles before topography, then the topography of the CPC and BMP-2@CPCB granules was observed by scanning electron microscopy (SEM, JSM-6360LV, JEOL, Japan).

### In-vivo investigation and histological evaluation

A total of six seven-week-old male C57 (C57BL/6) rats (17 ~ 19 g weight) were used for subcutaneous bone formation assessments. Rats were housed in steel hanging mesh cages, provided with a standard chow, and had no limited access to water. Experiments on rats and animal care were ethically approved by the Laboratory Animal Management and Welfare Ethical Review Committee of Zhejiang Chinese Medical University (ZSLL-2018-038) and ACTA Ethical Committee (202012). In this animal study, each rat was implanted with CPC or BMP-2@CPC at the left or right dorsal subcutaneous site randomly, which was consistence with a “split-mouth” design. Thus, for each kind of material, there were six sites for the following assessments (n = 6 per group, two dorsal subcutaneous sites per animal). General anesthesia (inhalation of isoflurane) was performed before surgery, and 0.25 g CPC or BMP-2@CPC granules were randomly implanted subcutaneously in the dorsal sites (simple cut and suture). For this purpose, rats were placed in a dorsal position and immobilized. The backs of the rats were shaved and disinfected. Dermal incisions were made in a longitudinal direction parallel to the spine. After implantation of CPC or BMP-2@CPC, interrupted sutures were used to close the skin.

Five weeks after the operation, the rats were sacrificed, and the implanted CPC and BMP-2@CPC granules samples were retrieved together with a minimum quantity of the surrounding tissue for chemical fixation and embedding, as previously reported [19]. With a systematic random-sampling strategy [20], the samples were sectioned into 7–9 slices of 600 μm thickness vertical to the short axis and with 1 mm intervals from each other. Of each sample, six slices were mounted on Plexiglas holders and then polished to 400 μm. The slices were then stained with McNeal’s Tetrachrome, basic Fuchsin, and Toluidine Blue O.

Biocompatibility of implanted CPC and BMP-2@CPC was evaluated based on the volume of fibrous capsule tissue and multinucleated giant cells (MNGC) volume density. In addition, osteoinductivity was assessed according to the density of bone and the osteoblast cells under the skin of rats. The total volume of the subcapsular space embraced by the fibrous connective tissue capsule was taken as a reference volume [21]. Within the fibrous capsule of each slice, the cross-sectional area of bone, fibrous connective tissue, and residual CPC were estimated using the Cavalieri/point-counting estimator technique [22]. The mean volume of bone, fibrous connective tissue, and residual CPC of each retrieved tissue of CPC or BMP-2@CPC groups were calculated by multiplying the sum of the cross-sectional area of bone, fibrous connective tissue, and residual CPC by the fixed distance between slices, defined as volume = (scale length / actual length × sieve length) ^ 2) × counted points × width of slice. The volume density of each tissue mentioned above was obtained by dividing the tissue volume by the total volume of the subcapsular space (reference volume).

**Fig. 1:**
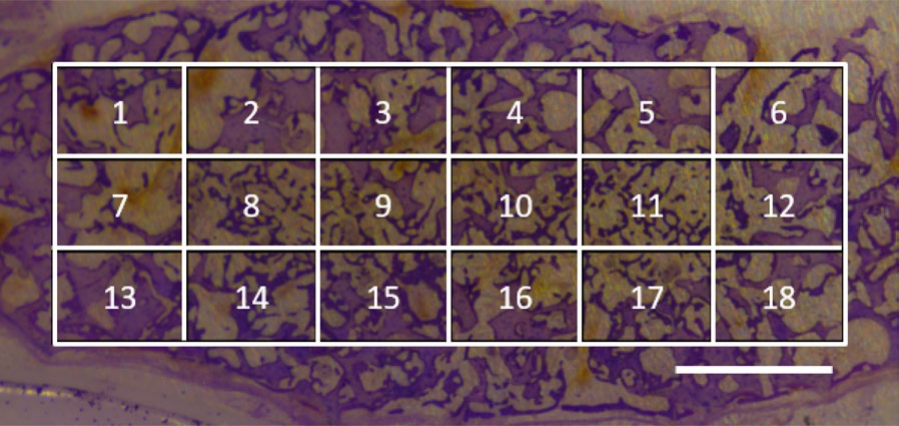
Randomization method for Cavalieri-counting for osteoblasts and MNGCs in each slice

Two small areas per slice were randomly selected to represent each slice's osteoblasts and MNGCs density (Fig. 1). Briefly, a grid was put on the slices to cover most of the tissue, high magnification photos at 200 × of two randomly selected areas were printed, and cell volume density was determined by a point-counting technique and calculated to represent the osteoblasts and MNGCs density [21]. A grid with 18 subareas is placed over the histology picture (scale bar, 2 mm), covering most of the tissue. Two random numbers will decide the randomly selected two subareas per slice. The two selected subareas will be analyzed in 200 × magnification, and the results from two randomly selected areas will represent this slice.

### Retrospective evaluation of BMP-2@CPC in maxillofacial surgery

Since there are no clinical evaluations of the short- and long-term adverse events of BMP-2@CPC in maxillofacial surgery, a retrospective descriptive study was carried out to analyze the potential adverse events of BMP-2@CPC in this clinical condition. Inclusion criteria were: (1) Oral facial bone disorders treated with sinus floor elevation, bone regeneration after cyst enucleation surgery, guided bone regeneration (GBR), alveolar ridge preservation and (2) complete clinical record in medical histories available. Exclusion criteria were: (1) Patients for whom the status at certain time points could not be verified by either the treating physician or any relative; (2) incomplete medical records; (3) isolated nasal bone fractures and dental fractures. All the associated data were collected from medical records from the Department of Maxillofacial Surgery in the Affiliated Dental Hospital of Jiamusi University. The used BMP-2@CPC in these maxillofacial operations is approved by the Chinese Food and Drug Administration (CFDA No. 2013: 34-60199), and the retrospective study protocol was approved by the ethical committee of the Academic Center for Dentistry Amsterdam (2020281). As the institute delivered the data that did not include the privacy information of the patients, the requirement for informed consent was waived.

**Fig. 2:**
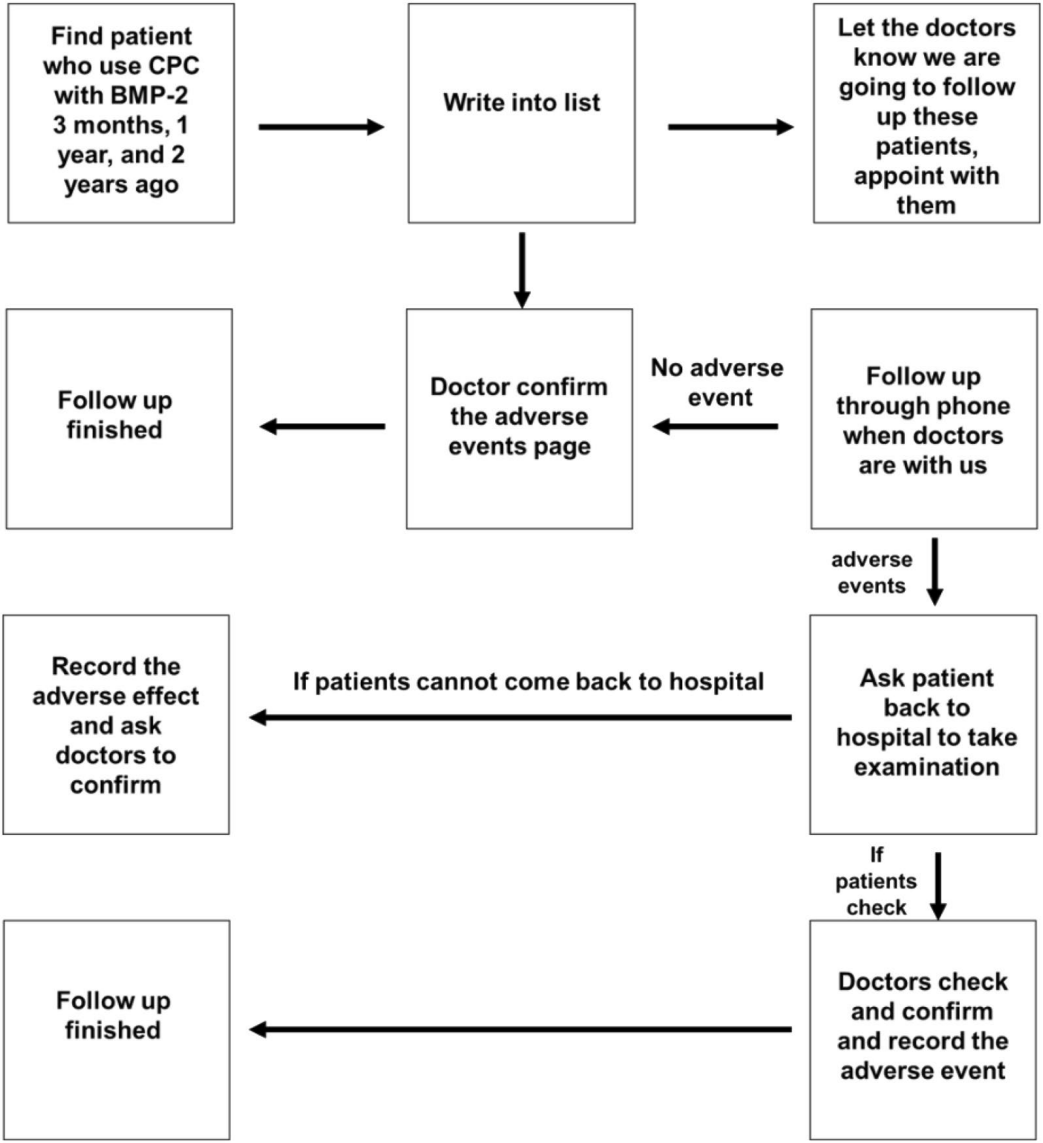
Workflow of this retrospective clinical study

The general workflow of this clinical study can be found in Fig. 2. For the inclusion criteria, subjects had to have BMP-2@CPC implantation in maxillofacial areas. The cut-off date of data collection was August 1, 2018, and all data were collected in a de-identified fashion and entered into a standardized case report form that included demographic information (gender, race, and age), diagnosis, surgical procedure, amount of implanted BMP-2@CPC, and any adverse events. Patients were followed up for up to 2 years.

For a short-term evaluation, the inflammation after surgery was detected using complete blood cell counting, mainly focused on the number of white blood cells (WBC) and neutrophil–lymphocyte-ratio (NLR). In addition, the patient’s body temperature, pain grade, and any self-reported adverse events were recorded.

## Statistical analysis

All data were represented as mean values with standard deviation (mean ± SD). Data were compared using the independent sample t-test and Fisher exact test with the significance level set at *p* < 0.05, using SPSS statistical software (version 26, IBM Corporation, NY, USA).

## Results

### Osteoinductivity and biocompatibility of BMP-2@CPC

#### Topography of CPC and BMP-2@CPC granules

**Fig. 3:**
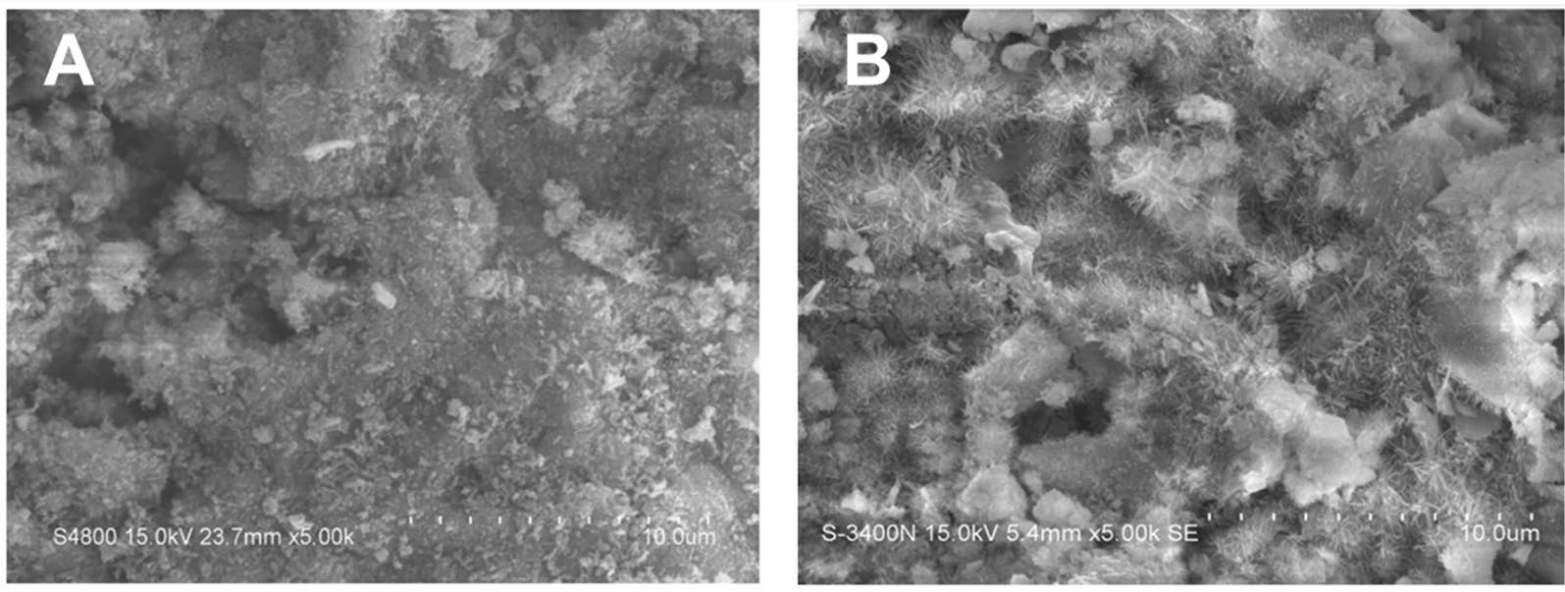
Surface topography of CPC (**A**) and BMP-2@CPC (**B**)

The surface of CPC and BMP-2@CPC granules were characterized by scanning electron microscope with a magnification of 5000 ×, as seen in Fig. 3. A rough globules-like structure with needle-like crystals covered on its surfaces was observed. The surface of the CPC granules in both groups looked similar, and the incorporation of BMP-2 in the BMP-2@CPC group did not visibly or significantly alter the surface topography of the granules in magnification 5000 ×.

#### Histological assessments

**Fig. 4:**
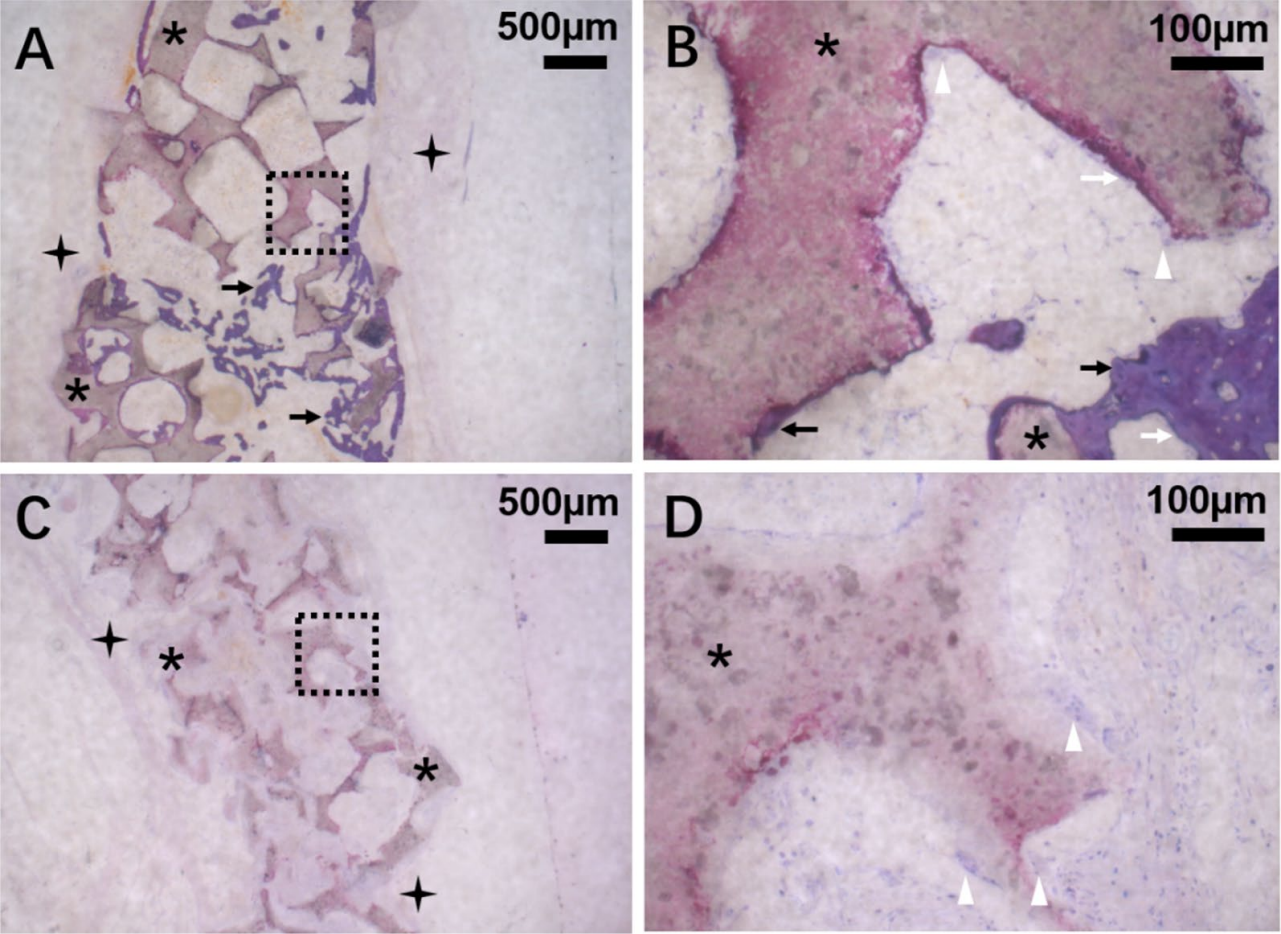
Representative light micrographs of sections from the CPC group and BMP-2@CPC group after 5 weeks of subcutaneous implantation in rats. In the BMP-2@CPC group (**A**), the newly formed bone (black arrow) directly deposited on the surface of CPC granules (asterisk) and formed a bone network between the CPC granules. **B** shows the magnification at 200 × of A (dashed box area), osteoblasts (white arrow) were observed on the surface of the newly formed bone, and MNGCs (white triangle) were in direct contact with the CPC surface. No purple-stained bone could be observed in the CPC group (**C**). **D** is the magnification at 200 × of C (dashed box area). MNGCs were observed on the surface of CPC granules. Fibrous connective tissue (four-pointed star) surrounded the implanted CPC and BMP-2@CPC samples

Five weeks after implantation in the ectopic model, the implanted CPC and BMP-2@CPC samples (consisting of granules) were surrounded by a fibrous connective tissue capsule. Bone tissue was observed on the surface of BMP-2@CPC granules and formed a bone network between the BMP-2@CPC granules (Fig. 4A). Osteoblasts were found to align in a characteristic epithelioid fashion on the surface of new bone (Fig. 4B). No bone tissue had been deposited on or around any of the CPC granules in the CPC group (Fig. 4C and D). MNGCs with relatively more considerable cell volume and characteristic multi-nuclei were found on the surface of CPC granules in CPC and BMP-2@CPC groups (Fig. 4B and D).

#### Histomorphometric assessments

**Fig. 5:**
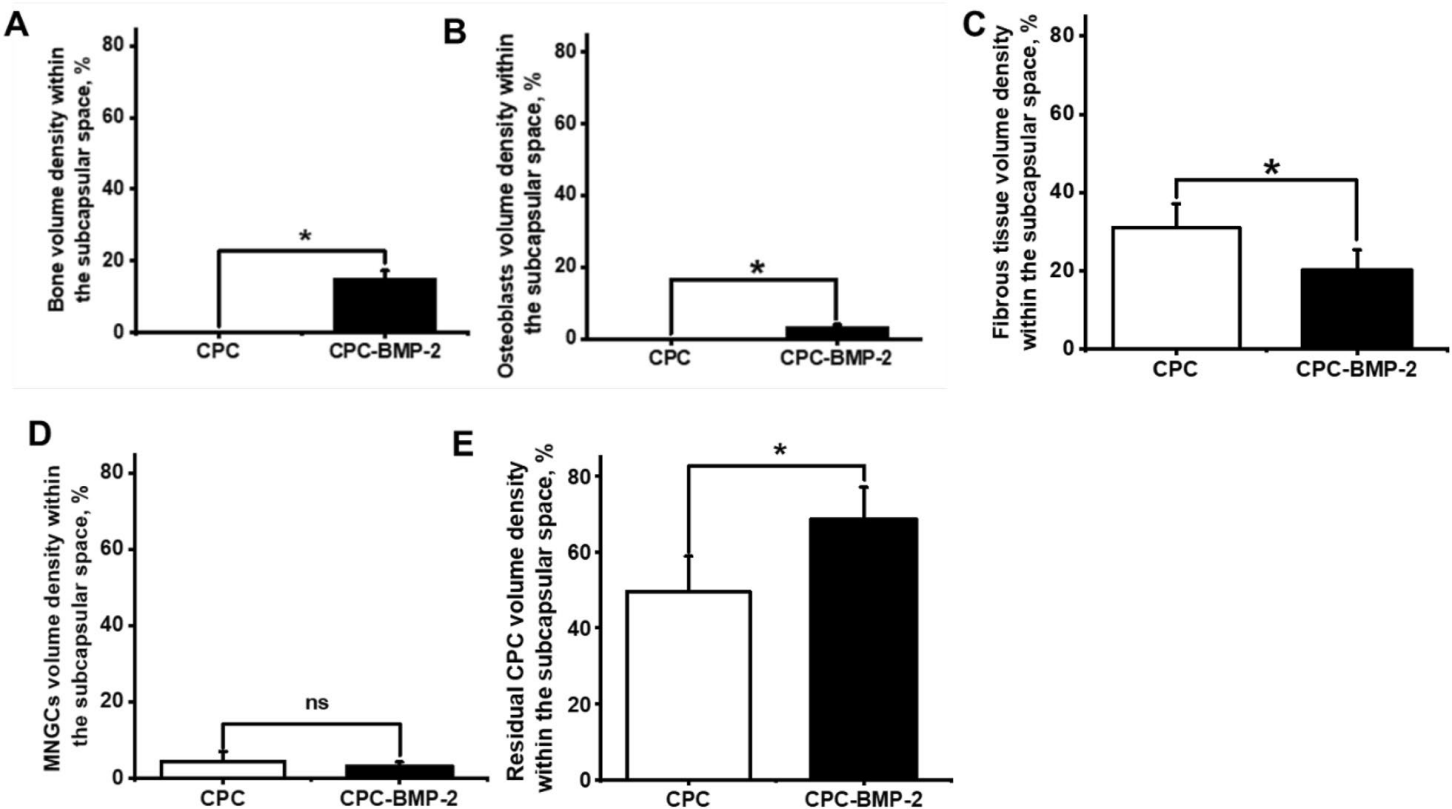
Bone formation and tissue foreign body reaction of BMP-2@CPC in rat’s subcutaneous sites. **A** Bone and **B** osteoblasts volume density of BMP-2@CPC and CPC groups. **C** Fibrous connective tissue, **D** MNGCs and residual volume density of CPC and BMP-2@CPC groups, **E** Residual volume density of materials of CPC and BMP-2@CPC groups. Data are shown as the mean percentage ± SD (n = 6) of the subcapsular space (reference volume) and showed significance (*) when compared between the groups, p < 0.05

After five weeks of subcutaneous implantation in rats, the osteoinductivity of CPC and BMP-2@CPC was gauged by estimating the volume density of bone (Fig. 5A) and osteoblasts density (Fig. 5B). Only the BMP-2@CPC group showed significantly higher bone volume density with 15 ± 2% (95% CI: 12%, 17%) and 0 ± 0% (95% CI: 0%, 0%) for the CPC group (*p* < 0.05, Fig. 5A), effect size 9.88. A difference was also found in osteoblasts density, the BMP-2@CPC group scored significantly higher with 3% ± 1% (95% CI: 2%, 4%), and 0% ± 0% (95% CI: 0%, 0%) for the CPC group, effect size 1.44 (*p* < 0.05, Fig. 5B).

The biocompatibility of CPC and BMP-2@CPC was evaluated by measuring the volume density of fibrous tissue (Fig. 4A and C, labeled with four-pointed star) and MNGCs (Fig. 4B and D, labeled with a white triangle) after five weeks of subcutaneous implantation in rats. Fibrous connective volume density of BMP-2@CPC group scored significantly lower with 20% ± 5% (95% CI: 12%, 29%) compared to 31% ± 6% (95% CI: 25%, 37%) in the CPC group effect size 1.99 (*p* = 0.026, Fig. 5C). No statistically significant difference in MNGCs density was found between the two groups, with 2% ± 1% (95% CI: 1%, 3%) in the BMP-2@CPC group and 3% ± 2% (95% CI: 1%, 5%) in the CPC group effect size 0.63 (*p* = 0.443), as shown in Fig. 5D.

The influence of the functionalization of BMP-2 on the biodegradability of CPC granules was also evaluated by measuring the residual CPC volume density (Fig. 4A and C) after five weeks of subcutaneous implantation in rats. A statistically significant difference was found for residual CPC and BMP-2@CPC volume density, of 50 ± 9% (95% CI: 35%, 64%) and 69% ± 8% (95% CI: 60%, 77%, effect size 2.23), respectively (*p* = 0.016, Fig. 5E).

### Retrospective description of clinical adverse events

#### General information on patients and surgery

The general information on the retrospective evaluation is presented in Table 1. A total of 110 patients were included in this retrospective study, which could be regarded as a postmarketing clinical follow-up study, and the age range of the patients is from 6 to 74 years old. Among these maxillofacial operations, impacted tooth extraction was the most, accounting for 72%; cystectomy, odontogenic tumor resection, and benign gingival tumor resection were 2% and 2%, respectively. For BMP-2@CPC applications, 65 patients received 0.5 g BMP-2@CPC (with 0.5 mg BMP-2) treatment, and the others were treated with 1 g BMP-2@CPC that contains 1 mg BMP-2. During the retrospection, only five adverse events were identified in four patients.

**Table 1:** Information on patients and surgery

	Number of patients	Number of adverse events	Fisher exact test
Gender	Male (53)	0	*p* = 0.12
	Female (57)	4	
Age (years) (20.1 ± 15.52)	6–12 (47)	0	*p* = 0.12
	13–18 (20)	1	
	19–50 (32)	2	
	51–74 (11)	1	
Surgery	Impact tooth removal (79)	1	*p* = 0.18
	Cystectomy (27)	3	
	Odontogenic tumor resection (2)	0	
	Benign gingiva tumor resection (2)	0	
Dose of BMP-2	0.5 mg (45)	0	*p* = 0.14
	1 mg (65)	4	
Adverse events		Swelling (2)	
		Bony mass (2)	
		Wound dehiscence (1)	

#### Postoperative situation

**Fig. 6:**
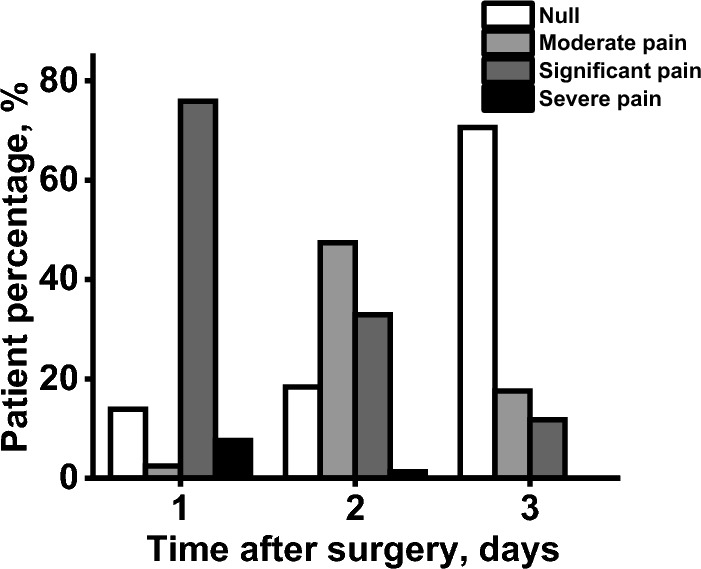
Post-surgery pain was assessed by a four-point verbal rating scale (VRS-4) in BMP-2@CPCimplantation in oral maxillofacial surgery

Short-term systemic inflammation was evaluated with the complete blood cell count (Table 2), body temperature, and pain level (Fig. 6). Table 2 shows the results of complete blood cell counts before and after BMP-2@CPC implantation of all patients. Based on the test, lymphocyte count significantly decreased from 2.6 to 2.2 after BMP-2@CPC implantation (*p* < 0.05), while white blood cells, neutrophils, and neutrophil–lymphocyte ratio (NLR) increased after implantation surgery. However, there were no significant differences in the regular reference interval. Similarly, the body temperature increased from 36.6 to 36.7 ℃, which was not statistically different. Meanwhile, as Fig. 6 showed, the post-surgical pain was also significantly decreased three days after surgery. These results indicated that the application of BMP-2@CPC in maxillofacial surgery was tolerant for the patients and did not provoke acute inflammation.

**Table 2:** Complete blood cell test

Parameters	Before implantation	95%CI	After implantation	95%CI	Effect size	*p*-value	Reference interval
White blood cells (10^9^/L)	7.3 ± 2.2	6.7, 8.0	9.8 ± 3.2	8.4, 10.4	0.88	0.25	6–20
Neutrophils (10^9^/L)	4.1 ± 1.8	3.6, 4.7	6.7 ± 3.1	5.4, 7.4	0.96	0.05	3–12
Lymphocytes (10^9^/L)	2.6 ± 0.9	2.3, 2.8	2.2 ± 0.8	1.9, 2.2	0.47	0.01	1–5
NLR	1.8 ± 1.1	1.5, 2.0	3.5 ± 2.1	2.7, 4.0	0.93	0.12	1–4

However, some mild (Grade 1) or moderate (Grade 2) adverse effects have also been identified in four patients. Two of these four patients suffered from mild and moderate bony mass, respectively, after BMP-2@CPC implantation. In addition, soft tissue mass and wound dehiscence were reported in 2 and 1 patients, respectively. Reassuringly, adverse events, including cancer, life-threatening cases, and death, were not reported after 24-month follow-up, and some patients even received dental implant treatment in the BMP-2@CPC filling area after the surgery. The radiographic images of this patient (Supplemental Fig. 3) were obtained later after requesting this information from the investigators.

## Discussion

Previous studies have shown that BMP-2 functionalized CPC is osteoinductive [9]. Consistent with these studies, our in vivo rat study also reveals that BMP-2@CPC is an osteoinductive bone substitute. Although BMP-2 endows CPC with osteoinductivity, its effect on the biocompatibility and biodegradability of CPC has not been well studied. In this study, BMP-2@CPC has significantly better biocompatibility than CPC. Meanwhile, both CPC and BMP-2@CPC are biodegradable bone substitutes, but BMP-2@CPC has significantly lower in vivo degradation than CPC. It is still unclear how BMP-2 inhibited the degradation of CPC. Based on the different topography, the altered geometrical morphology could be one of the reasons.

Foreign body reaction and fibrosis or fibrous capsule development are crucial chronic inflammatory and late-staged biological responses after material implantation [23]. MNGCs are formed by the macrophages found in copious amounts in human tissues, and MNGCs around biomaterials have long been regarded as a hallmark of a chronic inflammation process [24]. For this reason, MNGCs and fibrous tissue were assessed using histomorphometric analysis in the present study. BMP-2@CPC group has statistically significantly less fibrous tissue (*p* < 0.05) and significantly fewer MNGCs (*p* > 0.05) compared with the CPC group (Fig. 4D). This finding is consistent with our previous published data that the foreign-body reaction of calcium phosphate granules will be suppressed after BMP-2 functionalization [25, 26] as the presence of bone tissue will suppress the fibrous capsule [27].

In this study, decreased MNGCs volume density might be due to the elevated osteopontin. During bone regeneration, the level of osteopontin will elevate, and osteopontin is testified to suppress the fusion of macrophages into the formation of foreign body giant cells in vitro and in vivo [28]. Thus, the potential synergism may enhance the MNGC suppression.

BMP-2 released from BMP-2@CPC itself might also mediate the formation of MNGCs through immune regulation. A study has reported that BMP-2 can shift macrophage polarization: inhibiting the macrophage polarization to proinflammatory M1 and promoting wound healing M2-macrophages [29]. Moreover, degraded calcium phosphate is also responsible for the M2-macrophage polarization [30] due to a downregulation of inflammatory cytokines and upregulation of anti-inflammatory cytokines and as an essential participant during CPC degradation [31].

Besides biocompatibility, biodegradation, and osteoinduction of BMP-2@CPC, this study demonstrated the safety of BMP-2 @CPC used as filling material in maxillofacial surgery applications. Although few adverse events were identified in the retrospective evaluation, bone mass, and swelling are the major ones after BMP-2’s application in maxillofacial surgery, and a wound dehiscence case was also reported after benign gingival tumor removal. After retrieving this patient's medical records (NO.114), 1 g BMP-2@CPC was applied to the patient, and there was no infection at the incision site. Therefore, this dehiscence may be ascribed to the extensive tension caused by abundant filled CPC granules. In sum, after Fisher’s exact tests, all these adverse events reported in this study are not specific to gender, age, the dose of BMP-2@CPC, and the operations.

High-dose BMP-2 (> 2.1 mg/level) induced inflammation and soft tissue swelling have also been reported in cervical fusion patients [32]. Meanwhile, tissue inflammation, formation of bony shells, and increased osteoclast cell formation are associated with the high dose of BMP-2 [33]. This robust inflammation might be dose-dependently associated with releasing IL-6, IL-10, and TNF-α when high-dose BMP-2 was used [34]. Although the mechanism of how BMP-2 induces inflammation is still unclear, the consensus has been reached: a lower dose of BMP-2 is vital for clinical safety. In the retrospective study, we found that after applying 0.5 or 1 mg BMP-2 loaded into CPC in the maxillofacial application, only 5 adverse events in 4 out of 110 patients reported mild or moderate adverse events.

In general, these abovementioned adverse events were all dose-dependent. Compared to previous studies, less BMP-2 was used, leading to few mild and moderate adverse events, and all adverse events in this study occurred in patients who received 1 mg BMP-2 treatment. Compared with collagen sponges, the CPC carrier has a relatively closer composition and structure geometries of bone mineral and surface and slower degradation, making BMP-2@CPC more suitable and safer for clinical applications.

Only up to 24-month follow-up is one of the study's limitations since most implanted CPC was still not degraded. Further study will be scheduled for extended follow-up to evaluate the long-term clinical consequences. Besides, the potential disparate efficiency between various sources of BMP-2, such as CHO (Chinese hamster ovary)-cell-derived BMP-2 and Escherichia coli-derived BMP-2, loaded into CPC was not investigated. Although the lymphocytes statistically decreased after surgery (p < 0.05), the effect size is still smaller than 0.8, indicating that the effect size is not strong. This low effect size might be due to the limited sample size included in this study. Thus, a further study will give more insight into the potential different clinical performance of CPC with CHO-BMP-2 or *Escherichia coli*-BMP-2.

## Conclusion

BMP-2 loaded CPC is an osteoinductive bioceramic bone substitute with improved biocompatibility and decreased biodegradability compared to the CPC alone. In addition, based on the clinical medical records, BMP-2-loaded CPC also shows promising clinical safety in maxillofacial surgery.

## Supplementary Information

Supplementary file 1

## Data Availability

No datasets were generated or analysed during the current study.

## Abbreviations

BMP-2: Bone morphogenetic protein-2
CHO: Chinese hamster ovary
CPC: Calcium phosphate cement
MNGCs: Multinucleated giant cells
NLR: Neutrophil–lymphocyte-ratio
VRS: Verbal rating scale
WBC: White blood cell
