# Supplementary figures and images for: In vivo validation of osteoinductivity and biocompatibility of BMP-2 enriched calcium phosphate cement alongside retrospective description of its clinical adverse events
Source: Int J Implant Dent. 2024 Oct 30;10:47. doi: 10.1186/s40729-024-00567-6 (PMC11522231; doi:10.1186/s40729-024-00567-6)

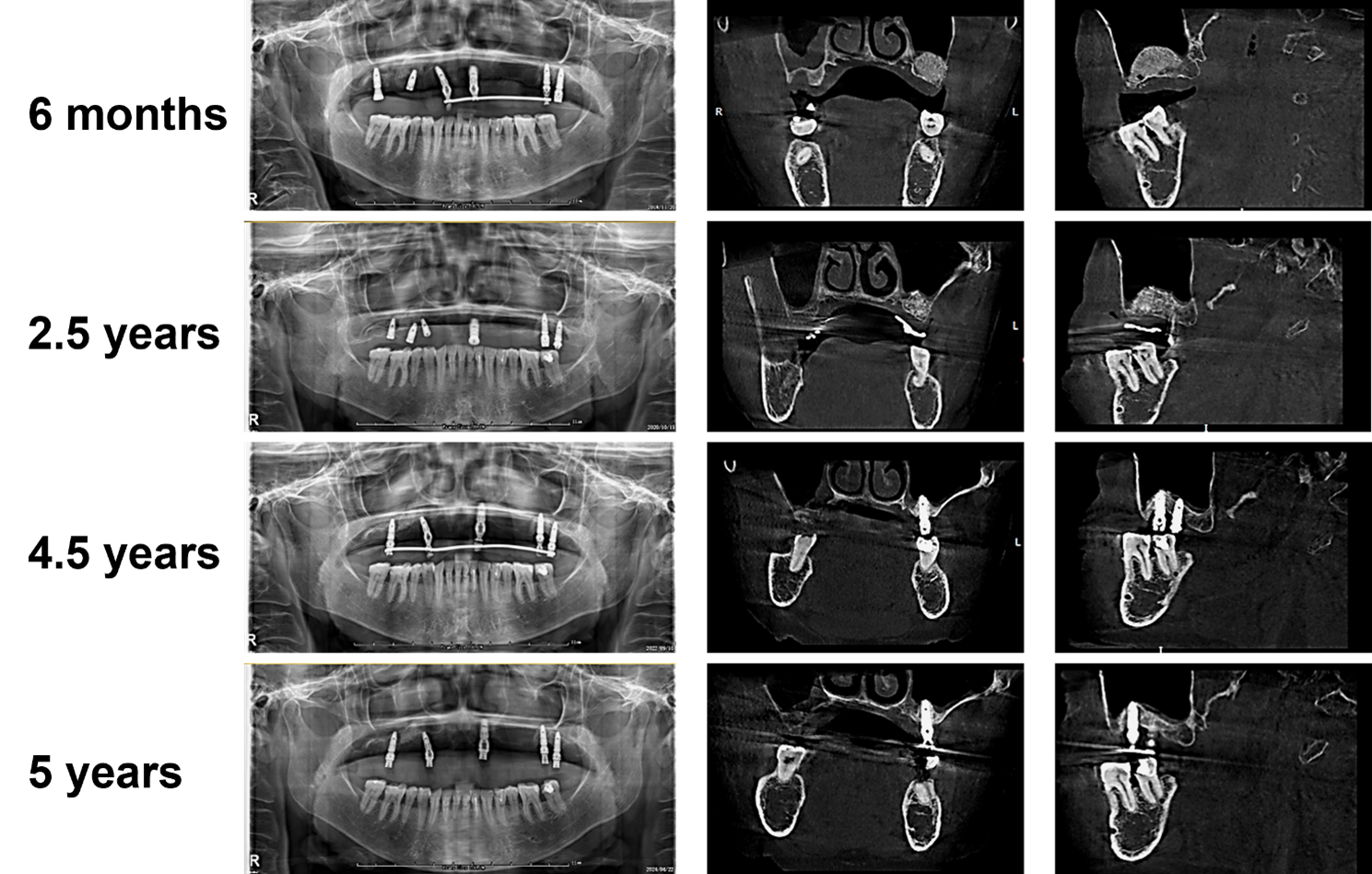

Supplement: Supplementary file 1 [file 40729_2024_567_MOESM1_ESM.tif]
